# Dual Enzymolysis Assisted by Acrylate or Phosphate Grafting: Influences on the Structural and Functional Properties of Jujube Residue Dietary Fiber

**DOI:** 10.3390/molecules29020478

**Published:** 2024-01-18

**Authors:** Yitao Zhao, Jianguo Xu, Yajun Zheng, Qi Li, Yihao Huang, Meichen Zong, Wangjin Guo

**Affiliations:** Food Science College, Shanxi Normal University, Taiyuan 030619, China; zhaoyitao30@163.com (Y.Z.); 18803470377@163.com (Q.L.); yourshyh@163.com (Y.H.); 15648635631@163.com (M.Z.); 19581580969@139.com (W.G.)

**Keywords:** jujube residue dietary fiber, dual enzymatic hydrolysis, phosphate grafting, acrylate grafting, functional capacities

## Abstract

Jujube residue is an abundant and low-cost dietary fiber resource, but its relatively lower hydration and functional properties limit its utilization as an ingredient of functional food. Thus, cellulase and hemicellulase hydrolysis, enzymatic hydrolysis assisted by phosphate grafting (EPG), and enzymatic hydrolysis assisted by acrylate grafting (EAG) were used to improve the functional properties of jujube residue dietary fiber (JRDF) in this study. The results evidenced that these modifications all increased the porosity of the microstructure of JRDF and increased the soluble fiber content, surface area, and hydration properties, but reduced its brightness (*p* < 0.05). Moreover, JRDF modified by enzymolysis combined with acrylate grafting offered the highest extractable polyphenol content, oil, sodium cholate, and nitrite ion sorption abilities. Meanwhile, JRDF modified via enzymolysis assisted by phosphate grafting showed the highest soluble fiber content (23.53 g∙100 g^−1^), water-retention ability (12.84 g∙g^−1^), viscosity (9.37 cP), water-swelling volume (10.80 mL∙g^−1^), and sorption ability of copper (II) and lead (II) ions. Alternatively, JRDF modified with cellulase hydrolysis alone exhibited the highest glucose adsorption capacity (21.9 g∙100 g^−1^) at pH 7.0. These results indicate that EPG is an effective way to improve the hypolipidemic effects of JRDF, while EAG is a good choice to enhance its hydration and hypoglycemic properties.

## 1. Introduction

Today, 537 million adults have diabetes worldwide, and there will be 1.31 billion diabetics by 2050 [1]. A main pathogeny for diabetes is a lack of dietary fiber (DF), and insufficient DF can also cause other serious harm to the body, including disorders of sugar and lipid metabolism, obesity, cardiovascular and cerebrovascular diseases, and colon cancer [2]. DF is a kind of polysaccharide that cannot be digested and absorbed by the human gastrointestinal tract. But some DFs can be fermented and utilized by microorganisms in the large intestine [3]. Recently, the role of DFs in prevention and adjuvant treatment of diabetes, hyperlipidemia, and hypertension has been evidenced in a large number of clinical trials [4]. Moreover, DFs are widely used in foods as thickening and water-retaining agents, texture improvers, emulsifiers, and clarifying agents [5]. In addition, DF is also available for use as a low-cost and renewable adsorbent in the purification of drinking water and the removal of contaminants (such as heavy metals, nitrite, and sulfides) from food [6]. Previous studies demonstrated that the hypolipidemic, hypoglycemic, and harmful-substance-removing activities of DFs were closely correlated with their microstructure, surface area, hydration properties, and adsorption capacities [6,7]. Some functional groups of DFs such as free hydroxyls, and the carboxyl, phenolic acid, and uronic acid groups improve their bounding affinity with water, oil, sugar, and metal ions, but phenolic and uronic acids are mainly in the form of conjugated mono-, di-, or oligosaccharides of fibers [8].

DF is classified as water insoluble (IDF) and soluble (SDF). Both IDF and SDF play an important role in the functional properties of DFs, and in an ideal DF, the SDF content could be no less than 30% [2]. Cereal and fruit byproducts such as rice bran, millet bran, and orange residue are potential supplements for human DF [5]. However, the application of cereal and fruit fibers as ingredients of functional foods is limited due to their low SDF (around 4 g∙100 g^−1^) and relatively poor hydration and adsorption properties [9]. Thus, certain physical, biological, and chemical methods such as alkaline treatment, high-pressure extrusion, ultrasonic treatment, enzymolysis, fermentation, and hydroxypropylation have been used to improve the hydration and adsorption properties of DFs [3,10,11]. Of these, phosphate and acrylate grafting are both shown to effectively improve the functional properties of starch and other fibers in food industry [5]. However, data regarding the composite effects of these methods, especially biological methods assisted by chemical ones on DFs, are scare.

Red jujube (*Ziziphus jujuba* Mill.) is a traditional species endemic to China. It has been cultivated in China for more than 4000 years [12]. Jujube residue is the main byproduct of jujube juice and offers a potential DF resource that is in abundant supply. Recently, jujube residue has received increasing attention because it contains a relatively high content of fiber (15 g∙100 g^−1^). However, the soluble fiber content is only around 5 g∙100 g^−1^ [13]. Cellulose and hemicellulose are the main components of jujube residue dietary fiber (JRDF), accounting for approximately 50.3 and 34.6 g∙100 g^−1^, respectively [14]. JRDF has a considerable water-retention capacity (around 4 g∙g^−1^), but its hydration properties and glucose, oil, and nitrite sorption capacities are poor [15,16,17]. Thus, the application of jujube residue as an ingredient in foods is rare. However, in recent decades, certain physical, chemical, and biological methods, along with acrylate grafting, have proven to effectively improve the physicochemical and functional properties of plant fibers [18,19,20]. To our best knowledge, there have been few studies on the synergistic influences of physical, chemical, and biological modifications on JRDF. Therefore, the purpose of this study was to investigate the influences of dual enzymatic hydrolysis assisted by acrylate grafting or phosphate grafting on the functional properties and the relevant structural, hydration, and adsorption properties of JRDF, with the aim to expand the potential applications of JRDF as as ingredient of functional foods.

## 2. Results and Discussion

### 2.1. Chemical Component of JRDF

The phosphate-grafting and acrylate-grafting degrees of JRDF-EPG and JRDF-EAG were 2.36 ± 0.33% and 3.57 ± 0.17%, respectively, demonstrating that phosphate and propenyl ester groups were introduced into the JRDF after enzymatic hydrolysis and phosphate grafting and acrylate grafting. As shown in Table 1, the three modifications (cellulase and hemicellulase hydrolysis alone, enzymolysis assisted by acrylate grafting, and enzymolysis assisted by phosphate grafting) showed no remarkable influences on the fat, protein, and ash contents of the JRDF (*p* > 0.05) but offered significantly improved SDF contents (*p* < 0.05), suggesting that these modifications all enhanced the hydrophilicity of the JRDF. In comparison, the IDF content of JRDF was obviously reduced after these modifications (*p* < 0.05), corresponding to its decreased cellulose, hemicellulose, and lignin contents. Cellulase and hemicellulase hydrolysis can crack the glucosidic bonds in cellulose and hemicellulose [19], exposing more hydrophilic groups and thereby reducing the IDF content of the JRDF.

Both JRDF-EPG and JRDF-EAG offered higher SDF contents than that of JRDF-E (*p* < 0.05), evidencing that enzymolysis assisted by phosphate grafting and acrylate grafting was more effective in improving the hydrophilicity of JRDF than enzymolysis alone. The phosphate and propenyl ester groups introduced after these modifications were also responsible for the higher SDF contents of JRDF-EPG and JRDF-EAG, respectively. The phosphate group was a polar group and had high hydrophilicity, while the propenyl ester group had both hydrophilicity and hydrophobicity [21]. JRDF-EPG showed a higher SDF content than JRDF-EAG (*p* < 0.05) because the introduced phosphate group had higher polarity than the propenyl ester group [18]. Furthermore, the SDF content of JRDF-EPG (23.53 g∙100 g^−1^) was similar to that of oat bran fiber (22.05 g∙100 g^−1^) [20], indicating its potential for applications as a functional food ingredient.

Additionally, JRDF-E, JRDF-EPG, and JRDF-EAG all showed higher extractable polyphenol contents than JRDF (*p* < 0.05), mainly due to the hydrolysis of cellulose by cellulase. Cellulase and hemicellulase caused the degradation of fibers and the release of more phenolic compounds [21,22], significantly surpassing the extractable phenol content of JRDF. JRDF-EAG showed the highest polyphenol content. Previous studies found that the introduced propenyl ester groups could increase the bounding polyphenol content of DFs [23], which may have been the case here.

### 2.2. Particle Sizes and Colors of JRDFs

As shown in Table 2, JRDF-E, JRDF-EAG, and JRDF-EPG all exhibited smaller particle sizes (D_3,2_: 59.38–95.23 μm) but larger surface areas (86.44–167.35 m^2^∙kg^−1^) than those of JRDF (*p* < 0.05). The main reason was that enzymatic hydrolysis can degrade glycosidic bonds and crack the cell well of JRDF [24], leading to a decrease in its particle size. Moreover, during phosphate grafting and acrylate grafting, heating and alkaline treatment can also cause the degradation of polysaccharide chains and thus an increased surface area of JRDF. JRDF-EAG showed the largest surface area (167.35 ± 4.42 m^2^∙kg^−1^) and the smallest particle size (59.38 ± 3.35 μm), followed by JRDF-EPG and JRDF-E. A bigger surface area indicates that the chance of DFs touching oil and water molecules is increased, which is conducive to the adsorption capacity of DFs [7].

Moreover, the L* value (representing brightness) of the JRDF was reduced while the a* and b* values (indicative of redness and yellowness, respectively) were both increased after these mixed modifications (*p* < 0.05), indicating that a browning reaction probably occurred in the JRDF. JRDF-EAG showed the highest Δ*E* (19.13, Table 2) among the modified JRDFs, likely because heating (70 °C) and sodium hydroxide (6.25 mol∙L^−1^) treatment during acrylate grafting can both cause a browning reaction for DFs [25]. However, a darker color may affect the JRDFs’ utilization in foods.

### 2.3. Structural Characteristics

#### 2.3.1. Surface Microstructure

**Figure 1:**
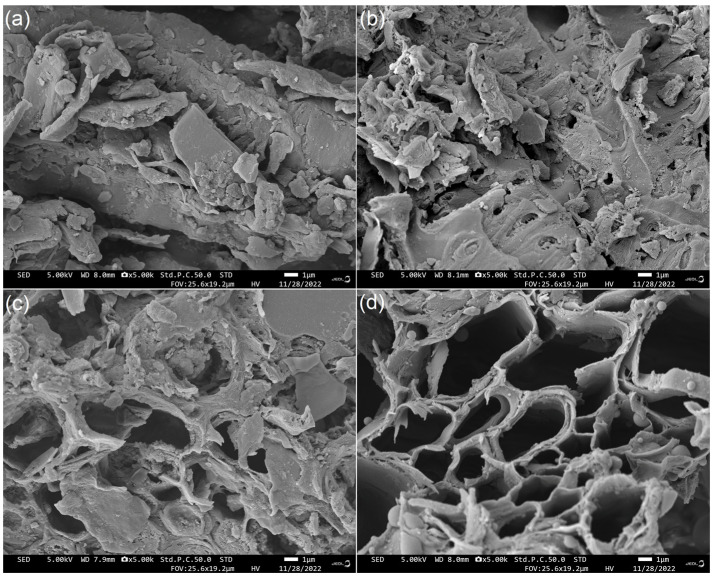
Scanning electron micrographs of JRDF (**a**), JRDF-E (**b**), JRDF-EAG (**c**), and JRDF-EPG (**d**) with a magnification of 5000× at 1 μm. JRDF, jujube residual dietary fiber; JRDF-E, JRDF modified with cellulase and hemicellulase hydrolysis; JRDF-EAG, JRDF modified with cellulase and hemicellulase hydrolysis assisted by acrylate grafting; and JRDF-EPG, JRDF modified with cellulase and hemicellulase hydrolysis assisted by phosphate grafting.

JRDF offered an irregular microstructure (Figure 1a), while JRDF-E, JRDF-EAG, and JRDF-EPG all showed microstructures with more fragmentation and pores (Figure 1b–d). After enzymolysis and chemical modifications, the polysaccharide chains and cell walls of the JRDF were destroyed, and chemical components such as fat, cellulose, and lignin were removed, resulting in more fragmented and porous microstructures [26]. A more porous and fragmented microstructure means DFs have a larger surface area and a higher sorption capacity in water and oil [27]. Our findings supported those of previous studies, which showed that enzymolysis, acrylate grafting, and hydroxypropylation can all make the microstructure of fibers more fragmented or porous [18,20].

#### 2.3.2. Fourier-Transformed Infrared Spectroscopy

**Figure 2:**
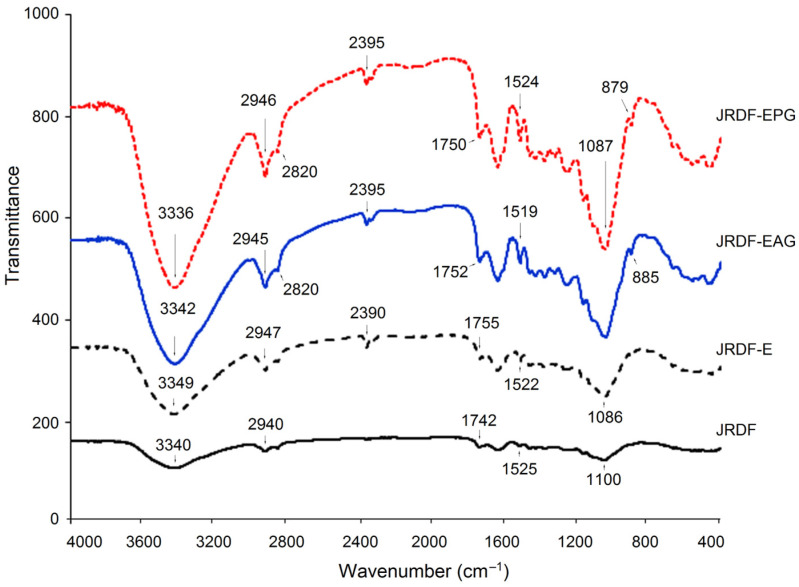
Fourier-transformed infrared spectra of JRDF, JRDF-E, JRDF-EAG, and JRDF-EPG.

Although the FT-IR spectrum of JRDF was similar to that of JRDF-E, JRDF-EAG, and JRDF-EPG, there were visible differences among them (Figure 2). The broad peak located at around 3340 cm^−1^ (representative of the stretching vibration of O–H) in the spectrum of JRDF moved to 3349, 3342, and 3336 cm^−1^ in the spectra of JRDF-E, JRDF-EAG, and JRDF-EPG, respectively, suggesting that the O–H bond of furfural acid was changed during these mixed modifications [28]. Compared to JRDF, a new peak at around 880 cm^−1^ (corresponding to the stretching vibration of the β-glycosidic bond) appeared in the spectra of JRDF-E, JRDF-EAG, and JRDF-EPG, evidencing the degradation of polysaccharide chains caused by ultrasound and enzymatic hydrolysis [29]. In the case of JRDF-EAG, the peaks at 1742 cm^−1^ (corresponding to asymmetric bending of the acetyl group) and 1100 cm^−1^ (representing the stretching vibration of C–O–C) transferred to 1752 and 1085 cm^−1^, respectively; and the peak at 1525 cm^−1^ (indicative of the stretching vibration of –C=O) transferred to 1519 cm^−1^, evidencing the introduction of the propenyl ester group [19]. Moreover, a new peak at around 2395 cm^−1^ (corresponding to the stretching vibration of the phosphate group) appeared in the spectrum of JRDF-EPG [10,30], demonstrating that the phosphate group had been grafted with JRDF. Similar results were obtained by Djordjević et al. [31]. In general, these results evidenced that the three modifications changed the chemical bonds of JRDF. However, the binding patterns and sites of the phosphate and acetyl groups with JRDF molecules require further investigation.

#### 2.3.3. X-ray Diffraction Profiles

**Figure 3:**
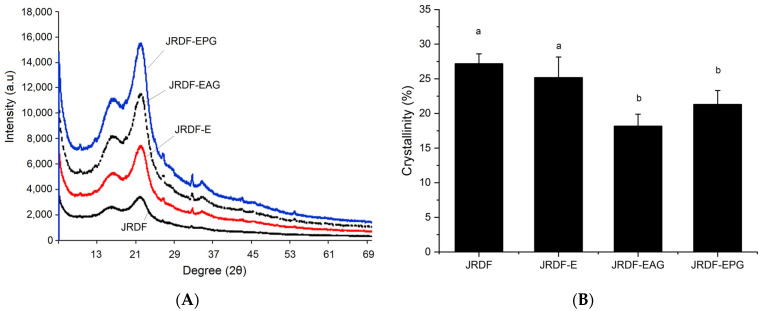
X-ray diffractions (**A**) and crystallinities (**B**) of JRDF, JRDF-E, JRDF-EAG, and JRDF-EPG. Different letters on the columns mean significant difference (*p* < 0.05).

As shown in Figure 3A, the JRDFs all offered a typical X-ray diffraction profile of fibers with two intensity peaks at 18.4° and 22.2° (corresponding to the cellulose crystal structures I and II, respectively) [31]. The crystallinities of JRDF-E, JRDF-EPG, and JRDF-EAG were lower than that of JRDF (*p* < 0.05), indicating that enzymatic hydrolysis combined with acrylate grafting or phosphate grafting destroyed the cellulose crystal structure of the JRDF. The crystal structure of the fibers was mainly maintained by hydrogen bonds formed between adjacent hydroxyl groups [10]. Cellulase and hemicellulase hydrolysis can both cause degradation of the glucosidic bonds of fibers, and the heating and alkaline treatments during acrylate and phosphate grafting can break down hydrogen bonds, leading to a lower crystallinity of JRDF-E, JRDF-EAG, and JRDF-EPG [19,32]. A similar trend was observed by other previous studies [31,33].

### 2.4. Hydration Properties

High hydration properties including the WRA, WSV, and viscosity allow fibers to rapidly interact with water molecules; expand their volume in water; and thus inhibit the diffusion of oil, heavy metal ions, or sugar in water or food, promoting the aggregation of these materials and facilitating the adsorption of DFs [34,35]. As shown in Table 2, JRDF-E, JRDF-EAG, and JRDF-EPG all exhibited a higher WRA, WSV, and viscosity than JRDF (*p* < 0.05), evidencing that dual enzymolysis alone or combined with acrylate grafting or phosphate grafting all effectively improved the hydration properties of the JRDF. The main reason was the higher soluble contents of JRDF-E, JRDF-EAG, and JRDF-EPG (Table 1). After enzymatic hydrolysis combined with acrylate grafting or phosphate grafting, the polarity of the JRDF was significantly increased, thereby improving the interactions between the JRDF and water molecules. Moreover, the more fragmented and porous surface microstructures and bigger surface areas of JRDF-EAG and JRDF-EPG (Figure 1c,d, Table 1) also contributed to their high WRA, viscosity, and WSV because a porous microstructure and bigger surface area are both conducive to the interactions between water and fibers [24]. Furthermore, the introduced propenyl ester and phosphate groups enhanced the steric hindrance between fiber chains [18], thereby improving the WSV of the JRDF.

JRDF-EPG showed the highest WSV (10.80 mL∙g^−1^), WRA (12.84 g∙g^−1^), and viscosity (9.37 cP) among all the JRDFs. After phosphate grafting, the introduced phosphate groups had high polarity and a strong affinity with water molecules [36], significantly increasing the viscosity, WSV, and WRA of JRDF. Since the polarity of the acrylate group was lower than that of the hydroxypropyl and phosphate groups, JRDF-EAG had a lower WSV and WRA than those of JRDF-EPG (*p* < 0.05). With that said, the viscosity of JRDF-EAG was higher than that of JRDF (*p* < 0.05); previous studies showed that introducing the acrylate group could enhance the resistance of fiber fluid and increase the viscosity of fibers [23]. In addition, the WSV, WRA, and viscosity of JRDF-EPG were all higher than those of JRDF-E (*p* < 0.05), suggesting that enzymolysis combined with phosphate grafting is a better choice to increase the hydration properties of JRDF than enzymolysis alone. Previous studies also showed that acetylation and phosphate grafting improved the hydration properties of millet fiber and corn starch and thus expanded their applications in the food industry [21,25].

### 2.5. Functional Properties

#### 2.5.1. Adsorption Capacity of Oil on JRDFs

As shown in Table 2, JRDF-EAG exhibited the highest OSA (3.73 ± 0.23 g∙g^−1^), followed by JRDF-EPG; meanwhile, JRDF-E showed a lower OSA than that of JRDF, although this difference was not significant (*p* > 0.05). After acrylate grafting, the introduced propenyl ester group significantly increased the affinity of JRDF-EAG for oil molecules [25]. Previous studies also found that esterification remarkably increased the OSA of DFs [18,37]. In addition, the high WSV (8.80 mL∙g^−1^), large surface area (167.35 m^2^∙kg^−1^, Table 2), and porous microstructure (Figure 1c) were all conducive to the OSA of JRDF-EAG. The higher WSV indicates that JRDF-EAG had a larger expansion volume in water, which increased the contact area between JRDF-EAG and oil [26]. Moreover, JRDF-EPG showed a higher OSA than JRDF (*p* < 0.05). One reason for this finding was the phosphate groups introduced after phosphate grafting, which could promote the formation of a network-like structure between polysaccharide chains, assisting JRDF’s adsorption of oil [18]. Additionally, other supporting factors were the high WSV and the porous microstructure of JRDF-EPG (Table 2 and Figure 1d). These results demonstrated that JRDFs adsorbed oil via chemical and physical adsorption.

#### 2.5.2. Sorption Ability of Sodium Cholate

Sodium cholate is an endogenous emulsifier and plays an important role in digestion and the absorption of lipid. Therefore, inhibiting the production of sodium cholate can effectively reduce the absorption of fat in the small intestine [3]. The results in Table 2 show that both JRDF-EAG and JRDF-EPG offered higher SASCs than that of JRDF (*p* < 0.05), which was in accordance with their higher OSA. The higher OSA indicated that the DFs had a stronger affinity with oil molecules. After acrylate grafting, the propenyl ester group that was introduced significantly increased the hydrophobicity of JRDF and thus enhanced its adsorbing ability for oil and sodium cholate. Moreover, the large surface area and porous microstructure contributed to the SASC of JRDF-EAG. In the case of JRDF-EPG, the phosphate group that was introduced increased the SDF content and hydrophilicity of JRDF, which was conducive to the affinity of DFs for sodium cholate, an endogenous emulsifier with hydrophobicity and hydrophilicity. Furthermore, the SASC of JRDF-E was lower than that of JRDF-EAG and JRDF-EPG (*p* < 0.05), demonstrating that acrylate grafting and phosphate grafting, rather than cellulase and hemicellulase hydrolysis, enhanced the SASC of JRDF. A similar trench was observed by Zheng et al. [18]. The high OSA and SASC of JRDF-EPG highlight its potential for application as an ingredient in hypolipidemic foods [35].

#### 2.5.3. Sorption Ability of Glucose

As was shown in Table 2, the GSAs of all JRDFs at pH 7.0 were higher than those at pH 2.0, indicating that JRDFs had a higher ability to adsorb glucose in the intestine than in the stomach because the acidic environment in the latter was not conducive to the dissociation of carboxylic and phenolic acid, which have a strong affinity for glucose [8]. JRDF-E and JRDF-EPG both offered higher GSAs (21.69–24.94 μmol∙g^−1^) than JRDF (*p* < 0.05), which can probably be attributed to their higher SDF contents and WSV, more porous microstructures, and larger surface areas (Table 1, Figure 1b–d, and Table 2). The higher WSV and larger surface areas indicate that the JRDFs had more of a chance to contact and adsorb glucose, while a porous microstructure meant that the JRDF had a higher ability to rupture glucose [34]. More importantly, the phosphate group that was introduced enhanced the hydrophilicity of JRDFs and thus improved their chemical adsorption of glucose. Accordingly, the GSAs of JRDF-E and JRDF-EPG were 2.45 and 2.13 times that of JRDF. Likewise, previous studies also found that enzymatic hydrolysis and phosphate grafting improved the GSAs of coconut cake fiber and Chia fiber [32,33]. In contrast, JRDF-EAG had a lower GSA than that of JRDF, although this difference was not significant (*p* > 0.05), highlighting the reducing minimal influence of acrylate grafting on the GSA of JRDF. The relatively high GSAs of JRDF-E and JRDF-EPG suggested their potential applications as ingredients of hypoglycemic foods [29].

#### 2.5.4. NISA

The JRDFs all exhibited higher NISAs at pH 2.0 than at pH 7.0, suggesting that nitrite ions were more easily adsorbed by JRDFs in the stomach. In an acidic environment, nitrite ions are easily transferred into nitrogen oxide, which can be easily adsorbed by organic acids in DFs [27]. JRDF-E, JRDF-EPG, and JRDF-EAG all exhibited higher NISAs (11.43–21.64 μmol∙g^−1^, Table 2) than that of JRDF (*p* < 0.05), mainly due to their higher phenolic acid contents (Table 1). JRDF-EAG offered the highest NISA (21.64 ± 1.95 μmol∙g^−1^) at pH 2.0, corresponding to it having the highest phenol content (Table 1). Moreover, the more porous microstructures (Figure 1b–d), larger WSVs, and smaller particle sizes (Table 2) all indicate that these modified JRDFs had a higher capacity to adsorb nitrite ions [34]. Additionally, the phosphate and propenyl ester groups that were introduced both had a relatively strong affinity for nitrite ions [18].

#### 2.5.5. Copper Ion (II) Sorption Ability

**Figure 4:**
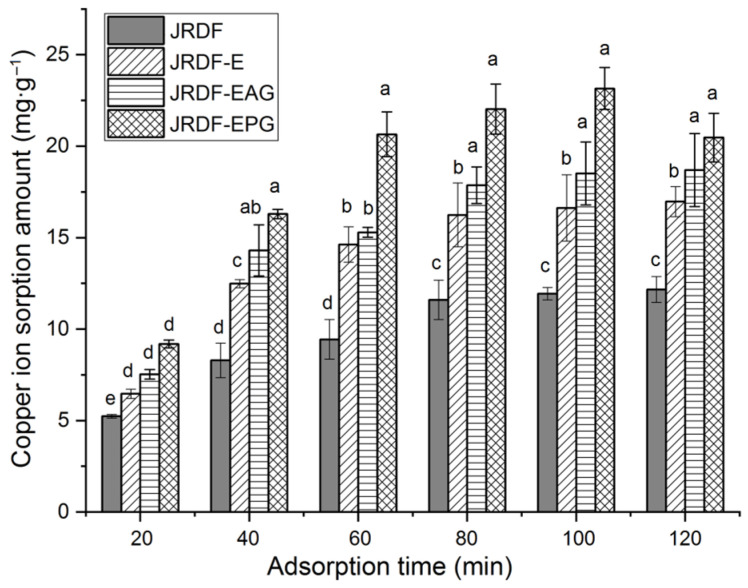
Isothermal adsorption kinetics of copper ions (II) on JRDF, JRDF-E, JRDF-EAG, and JRDF-EPG. Different lowercase letters (a–e) on the bars evidence the difference is significant (*p* < 0.05).

Excess copper ions can cause damage to the liver and kidneys [38]. Figure 4 depicts the isotherm adsorption kinetics of copper ions (II) on JRDFs. JRDF, JRDF-E, JRDF-EAG, and JRDF-EPG all showed a time-dependent Cu^2+^ sorption capacity, reaching an equilibrium sorption amount at around 80 min. Furthermore, JRDF-E, JRDF-EAG, and JRDF-EPG all showed higher ESCIs than that of JRDF (*p* < 0.05) (Table 2), evidencing that enzymatic hydrolysis alone or with phosphate grafting or acrylate grafting increases the adsorption ability of copper ions on JRDF. One reason for this is that enzymolysis causes the degradation of cellulose and lignin and thus the release of more functional groups such as uronic, carboxyl, and phenolic groups, which have a relatively strong affinity with copper ions [26]. In addition, the phosphate and ester groups that were introduced remarkably increased the negative charges of the JRDFs [18], thus increasing their ESCIs. JRDF-EPG exhibited the highest ESCI (24.14 ± 0.27 mg∙g^−1^) because the introduced phosphate group has a strong chelating ability with copper ions [36]. Meanwhile, the ESCIs of JRDF-EAG and JRDF-EPG were higher than that of JRDF-E (*p* < 0.05), evidencing that enzymolysis assisted by phosphate grafting or acrylate grafting was more effective at increasing the ESCI of JRDF than enzymolysis alone.

#### 2.5.6. Lead Ion (II) Sorption Capacity

**Figure 5:**
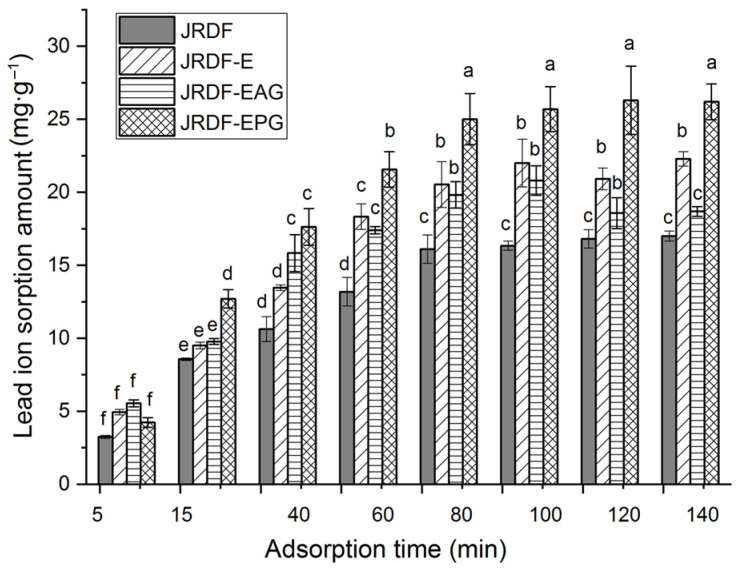
Isothermal adsorption kinetics of lead ions (II) on JRDF, JRDF-E, JRDF-EAG, and JRDF-EPG. Different lowercase letters (a–f) on the bars evidence that the difference is significant (*p* < 0.05).

Lead pollution can damage the human nervous system and induce mania, coma, and even death [27]. As shown in Figure 5, JRDF, JRDF-E, JRDF-EAG, and JRDF-EPG all showed a time-dependent sorption of lead ions (II) within 5–80 min and reached an equilibrium sorption value at around 65 min. Moreover, the ESLIs of JRDF-E, JRDF-EAG, and JRDF-EPG were higher than that of JRDF (*p* < 0.05, Table 2), mainly due to their larger WSV and surface area (Table 2), more porous microstructure (Figure 1b–d), and higher polyphenol contents (Table 1), because a larger WSV, larger surface area, and porous microstructure indicate that the fibers had a greater chance and capacity to capture lead ions [35]. In addition, the phosphate and propenyl ester groups that were introduced all increased the negative charge of JRDF, thereby improving its ESLI. JRDF-EPG showed the highest ESLI (25.37 ± 0.32 mg∙g^−1^), which can mainly be attributed to the high metal chelation ability of the introduced phosphate group [36]. The ESLIs of JRDF-EAG and JRDF-EPG (20.45–25.37 mg∙g^−1^) were higher than those of acrylate-grafted banana fiber (10.45 mg∙g^−1^) [23], corn starch (15.32 mg∙g^−1^) [27], and palm kernel fiber (19.8 mg∙g^−1^) [38] but lower than that of activated carbon (51.15 mg∙g^−1^) [31], suggesting their potential applications to remove heavy metals from foods.

## 3. Materials and Methods

### 3.1. Materials and Reagents

Craw jujube residue was donated by Sanduo Juice Co., Ltd., Yushe, China. Hemicellulase (2.0 × 10^4^ U·g^−1^), cellulase (Trichoderma Vride G, 3.0 × 10^5^ U·g^−1^), amyloglucosidase (Aspergillus niger, 1.0 × 10^5^ U·g^−1^), and α-amylase (Bacillus licheniformis) were all purchased from Yeyan Biotechnology Corporation (Shuzhou, China). Sodium sulfate, acrylic acidisopropenyl, sodium hydroxide, and other analytical reagents were purchased from Shanghuiyuan Biotech (Shijiazhuang, China).

### 3.2. Preparation of JRDF

Briefly, craw jujube residue was dried, ground, and sieved using a ZJ-IIC sieve (with an aperture of 150 μm, Zhuji Sieve Factory, Zhuji, China) [6]. The jujube residue powder (18 g) was dispersed in deionized water (dH_2_O, 360 mL) and then hydrolyzed by 0.2 g of α-amylase (at pH 7.0 and 65 °C for 90 min), 0.2 g of papain (at pH 7.0 and 50 °C for 120 min), and 0.15 g of glucoamylase (at pH 4.0 and 60 °C for 120 min) in sequence. After heating at 100 °C for 12 min, the mixture was filtered, and the residue on the filter paper was collected and dried (50 °C, 3 h) to obtain jujube residue dietary fiber (JRDF).

### 3.3. Dual Enzymatic Hydrolysis of JRDF

JRDF (20 g) and 260 mL of phosphate buffer (pH 4.5, 0.1 mol∙L^−1^) were added into a glass flask and mixed thoroughly [33]. Thirty minutes later, cellulase (75 U∙g^−1^) was added, and the flask was shaken using an EZC-004H water bath oscillator (Shanyu Oscillator Factory, Nantong, China) with a temperature of 55 °C and shaking rate of 195 r∙min^−1^. Two hours later, the mixture was adjusted to pH 6.5 with the addition of NaOH (0.1 mol∙L^−1^) and hemicellulase (25 U∙g^−1^ JRDF). The reaction was continued at a temperature of 35.0 °C and shaking rate of 195 r∙min^−1^ for 2 h. Then, the mixture was adjusted to pH 7.0 and heated at 100 °C for 5 min. After filtration, the residue was dehydrated using a GFGZ-II dryer (Huaxing Inst. Fac., Hengyang, China) with a heating temperature of 50 °C. Six hours later, jujube residue dietary fiber treated with cellulase and hemicellulase hydrolysis (JRDF-E) was obtained.

### 3.4. Acrylate Grafting of JRDF-E

Sodium hydroxide (6.25 mol∙L^−1^, 24 mL), dH_2_O (180 mL), and acrylic acid (15 mL) were mixed in a triangular flask and shaken in the EZC-004H oscillator at 65 r∙min^−1^ and 25 °C [23]. Forty minutes later, 2 g of JRDF-UE was added, and suspension was continuously shaken at 65 r∙min^−1^ for ten minutes. Next, thiosalicylic acid (58.4 mmol∙L^−1^, 1.5 mL) and potassium thiosulfate (74 mmol∙L^−1^, 2.25 mL) were added in sequence. The reaction mixture was shaken at 195 r∙min^−1^ and 70 °C. Three hours later, the suspension was filtered through a nylon sieve with an aperture of 100 μm, and the residue was washed using anhydrous ethanol (30 mL) in triplicate. After heating at 50 °C for 8 h, JRDF modified via enzymolysis assisted by acrylate grafting (JRDF-EAG) was obtained. The grafting degree was determined by following the same procedures as Rani et al. [23].

### 3.5. Phosphate Grafting of JRDF-E

JRDF-E (25 g) was dispersed in 250 mL of dH_2_O and stirred in the EZC-004H oscillator at 145 r∙min^−1^ and 25 °C [18]. Fifteen minutes later, the suspension was adjusted to pH 11, and then 3 g sodium trimetaphosphate and sodium tripolyphosphate (0.3 g) were added. Next, the reaction suspension was shaken at 145 r∙min^−1^ and 47 °C in the oscillator. Three hours later, the suspension was adjusted to pH 7.0 and then filtered with Waterman filter paper. The residue was rinsed with dH_2_O in triplicates (20 mL every time) and dried in the DHG-9053A blast drying oven with a heating temperature of 50 °C. Six hours later, JRDF modified via enzymolysis assisted by phosphate grafting (JRDF-EPG) was obtained. The grafting degree was defined as the increment in phosphorus content in JRDF-EPG after phosphate grafting [39].

### 3.6. Chemical Composition Measurement

The moisture, fat, protein, and ash contents of JRDF, JRDF-E, JRDF-EPG, and JRDF-EAG were separately determined using the methods AOAC.920.39, AOAC.92.05, AOAC.955.04, and AOAC.924.05 (AOAC, 2000). The acid-insoluble lignin, neural detergent fiber, and acid detergent fiber were measured as per the procedure from Souza et al. [40] to calculate the lignin, cellulose, and hemicellulose contents, respectively. Moreover, the total dietary fiber (TDF) and IDF contents were determined using the AOAC.991.43 method (AOAC, 2000), and the SDF content was calculated from them [11].

### 3.7. Particle Size Analysis

A nanometer laser particle size analyzer (WINNER 2309A, Jinan Winner particle Technology Co., Ltd., Jinan, China) with a refractive index of 1.33 was employed to determine the D_3,2_ (Sauter mean diameter, μm) and specific surface area of JRDFs [18].

### 3.8. Color Analysis

Color indexes including *L* (the lightness of DF), *b* (the redness), and *a* (the yellowness) values were measured with an NH810 spectrocolorimeter (Three-NH Colorimeter Co., Shenzhen, China) with D65 light source. The color difference (Δ*E*) was calculated with untreated JRDF as the control:

(1) $$\Delta E=\sqrt{{\left(L-L_{0}\right)}^{2}+{\left(a-a_{0}\right)}^{2}+{\left(b-b_{0}\right)}^{2\;}}\;$$

where *L*_0_, *a*_0_, and *b*_0_ are the color indexes of the untreated JRDF.

### 3.9. Structural Characteristics

#### 3.9.1. Surface Microstructure Analysis

Approximately 2 mg of JRDFs was placed on a preparation table and coated with a 10 nm gold layer [22]. A JEOL-JSM-7500F scanning electron microscope (Japan Electronics Co., Ltd., Showima city, Japan) was used to scan the microstructures of the samples with an acceleration voltage of 10 kV. Micrographs were captured at a magnification of 2000× and scale bar of 5 μm.

#### 3.9.2. Fourier-Transformed Infrared Spectroscopy (FT-IR)

An FT-752 FT-IR spectrometer (Jinke Instrument Factory, Shanghai, China) was employed for FT-IR analysis with a scanning range of 4000–400 cm^−1^, according to the same procedures used by Zhang et al. [22].

#### 3.9.3. X-ray Diffraction Investigation

An X-ray diffraction investigation was performed on an Ultima IV-185 X-ray powder diffractometer (Rigaku Corporation of Japan, Tokyo, Japan) according to the procedures used by Liu et al. [24]. The difference between the lowest and the maximum intensities per the maximum intensity was defined as the crystallinity (%) of a sample.

### 3.10. Hydration Properties Analysis

Hydration properties including the water-retaining ability (WRA) and water-swelling volume (WSV) of JRDFs were determined according to the methods used by Zheng et al. [18].

### 3.11. Viscosity

Viscosity determination was administrated on a RAV-I5 viscometer (Yebei Viscosity Instrument Factory, Zhuhai, China). The concentration of JRDF was 2.5 g 100 mL, and the shear rate was 600 s^−1^.

### 3.12. Functional Properties

#### 3.12.1. Oil Sorption Ability

The soy oil sorption ability (OSA) of JRDFs was conducted according to the same procedures from Zheng and Li [33]. Briefly, JRDFs (1 g) were mixed with soybean oil in a centrifugal tube and left for 1 h at 25 °C. The mixture was then centrifuged at 1500× *g* for 10 min, the supernatant decanted, and the pellet was recovered by filtering through a linen mesh. OSA is expressed as follows:

(2) $$ORA\;(g/g)=\left(pellet\;weight-dry\;weight\right)/dry\;weight$$

#### 3.12.2. Sodium Cholate Sorption Ability

The sodium cholate sorption ability (SCSA) of JRDFs was conducted according to the same procedures from Peerajit et al. [41]. Briefly, JRDFs (2 g) were mixed with 100 mL of sodium cholate (1 mg∙mL^−1^, pH 7.0) and incubated in a shaking water bath at 37 °C for 2 h. Afterward, the suspension was centrifuged at 3000× *g* for 10 min, and then 1 mL of mixture was collected and mixed with 6 mL of 45% sulfuric acid and 1 mL of 0.3% furaldehyde. After incubation at 65 °C for 30 min, the absorbance at 620 nm was measured. SCSA was calculated as follows:

(3) $$SCSA\left(g\cdot g^{-1}\right)=(C_{0}-C_{t})\times V/W$$

where *C_t_* and *C*_0_ were the sodium cholate concentrations of the reaction solution (1 mg∙mL^−1^) before and after the adsorption, respectively; *V* is the volume of reaction solution; and *W* is the weight of JRDFs.

#### 3.12.3. Glucose Sorption Ability

In conical flasks, JRDFs (1 g) were dispersed in 100 mL of glucose solutions with different concentrations (0.1, 0.2, 0.3, 0.4, and 0.5 g∙L^−1^) and then adjusted to pH 2.0 or pH 7.0, respectively [18]. The suspensions in the flasks were shaken at 25 °C in the EZC-004H oscillator with a shaking rate of 145 r∙min^−1^. Three hours later, the suspensions were filtered through a nylon cloth via aperture of 100 mesh. The filtrate liquid was used for glucose content determination with the phenol–sulfuric acid method [42]. The glucose sorption ability (GSA) is defined as follows:

(4) $$GSA\;\left(mg\cdot g^{-1}\right)=\left(C_{0}-\left.C_{S}\right.\right)\times V/\;W$$

where *V*, *W*, *C*_0_, and *C_S_* are the glucose solution volume, dry weight of JRDFs, and glucose concentrations before and after adsorption, respectively.

#### 3.12.4. Nitrite Ions’ Sorption Ability

One gram of JRDFs was dispersed in 200 mL of NaNO_2_ (2 μg∙mL^−1^) and adjusted to pH 2.0 or 7.0 with acetic acid (0.25 mol∙L^−1^); then, they were shaken in the EZC-004H oscillator at 135 r∙min^−1^ and 35 °C for 120 min [18]. Next, the suspensions were filtered, and the filtrate liquid was collected. The N-1-naphthylethylenediamine dihydrochloride method was used for the nitrite concentration determination [29]. After adsorption, the reduction in nitrate concentration according to the weight of JRDFs was defined as the nitrite ion sorption ability (NISA).

#### 3.12.5. Copper Ion (II) Sorption Capacity

JRDFs (0.5 g) were dispersed in 125 mL of CuSO_4_ (325 μg∙mL^−1^) and then shaken at 165 r∙min^−1^ and 25 °C for 10, 20, 40, 60, 80, and 100 min in the EZC-004H oscillator [18]. Next, the suspension was filtered through a nylon cloth (120 mesh). The filtrate liquid was pooled and used for copper ion (II) concentration determination with the KI–soluble starch titration method [43]. The equilibrium sorption of copper ions (II) (ESCI) on JRDFs was calculated as follows:

(5) $$ESCI\;\left(mg\cdot g^{-1}\right)=125\times \left(C_{0}-C_{S}\right)/\;W$$

where *W*, *C*_0_, and *C_S_* are the dry weight of JRDFs, initial Cu^2+^ concentration, and Cu^2+^ concentration after the adsorption, respectively.

#### 3.12.6. Lead Ion (II) Sorption Ability

Isothermal adsorption kinetics of lead ions (Pb^2+^) on JRDFs was investigated according to the modified procedures from Rani et al. [23]. Briefly, JRDFs (1 g) were dispersed in 220 mL of lead nitrate (70 mg∙L^−1^) and then shaken in the EZC-004H oscillator at 175 r∙min^−1^ and 35 °C for 5, 20, 35, 50, 65, 80, 95, and 110 min. Next, the suspensions were filtered using a nylon cloth (120 mesh), and then the filtrate liquid was used for Pb^2+^ concentration determination with the flame atomic absorption spectrometry method [27]. The equilibrium sorption of lead ions (II) (ESLI) on JRDFs was calculated as follows:

(6) $$ECLI\;\left(mg\cdot g^{-1}\right)=(C_{0}-C_{S})\times V/M$$

where *V*, *W*, *C*_0_, and *C_S_* are the Pb(NO_3_)_2_ solution volume, dry weight of JRDFs, and the initial and final Pb^2+^ concentrations, respectively.

### 3.13. Statistical Analysis

Each test was performed at least three times. Statistical analysis was performed using the SPSS program (Ver.24.0, SPSS Inc., Chicago, IL, USA), and Duncan’s multiple comparison was used for analysis of variance between data, with a significance level of 95% (*p* < 0.05).

## 4. Conclusions

Dual enzymatic (cellulase and hemicellulase) hydrolysis alone and enzymatic hydrolysis separately combined with phosphate grafting and acrylate grafting all made the microstructure of JRDF more porous and increased the SDF content, surface area, and hydration properties of JRDF (*p* < 0.05). Enzymolysis combined with acrylate grafting significantly improved the polyphenol content and hydrophobicity of JRDF and thus enhanced its oil, sodium cholate, and nitrite ion sorption abilities. Meanwhile, enzymolysis assisted by phosphate grafting remarkably improving the hydrophilicity of JRDF, resulting in higher SDF content; viscosity; and glucose, copper, and lead ion sorption amounts of JRDF (*p* < 0.05). Therefore, dual enzymolysis assisted by phosphate grafting is an effective way to expand the application of JRDF as an ingredient of hypoglycemic foods or a heavy-metal adsorbent, while dual enzymolysis assisted by acrylate grafting can expand the applications of JRDF as an ingredient of hypolipidemic foods. However, further work is required to improve our understanding of the in vitro functional properties of these modified JRDFs.

## Figures and Tables

**Table 1:** Compositions of jujube residue dietary fiber (JRDF), JRDF treated with cellulase and hemicellulase hydrolysis (JRDF-E), and JRDF modified via enzymolysis assisted by phosphate grafting (JRDF-EPG) or acrylate grafting (JRDF-EAG).

Constituent	Jujube Residue	JRDF	JRDF-E	JRDF-EAG	JRDF-EPG
Moisture (g∙100 g^−1^)	6.34 ± 0.36 a	7.47 ± 0.20 a	5.38 ± 0.19 a	5.99 ± 0.24 a	6.46 ± 0.17 a
Fat (g∙100 g^−1^)	2.07 ± 0.09 a	1.28 ± 0.09 b	1.54 ± 0.02 b	1.12 ± 0.09 b	2.08 ± 0.09 a
Ash (g∙100 g^−1^)	1.72 ± 0.07 a	2.19 ± 0.21 a	1.86 ± 0.08 a	2.42 ± 0.09 a	2.05 ± 0.22 a
Protein (g∙100 g^−1^)	1.94 ± 0.09 a	1.38 ± 0.08 a	1.39 ± 0.07 a	1.74 ± 0.08 a	1.89 ± 0.09 a
Total dietary fiber (g∙100 g^−1^)	18.23 ± 1.37 b	75.38 ± 2.28 a	81.37 ± 2.84 a	80.93 ± 4.82 a	80.09 ± 3.58 a
Insoluble dietary fiber (g∙100 g^−1^)	14.92 ± 0.32 d	69.72 ± 4.52 a	66.43 ± 3.34 a	62.95 ± 1.79 b	56.56 ± 4.95 c
Soluble dietary fiber (g∙100 g^−1^)	3.31 ± 0.08 e	5.66 ± 0.11 d	14.94 ± 1.18 c	17.98 ± 2.56 b	23.53 ± 2.32 a
Extractable phenols (g∙100 g^−1^)	0.76 ± 0.05 d	0.83 ± 0.01 c	1.14 ± 0.08 b	1.56 ± 0.11 a	1.23 ± 0.02 b
Cellulose (g∙100 g^−1^)	51.13 ± 3.64 a	53.56 ± 3.47 a	42.19 ± 3.25 b	39.38 ± 2.77 b	40.99 ± 3.67 b
Lignin (g∙100 g^−1^)	12.52 ± 1.33 a	12.53 ± 0.55 a	9.85 ± 0.08 a	10.79 ± 0.34 a	10.73 ± 1.05 a
Hemicellulose (g∙100 g^−1^)	33.54 ± 0.45 a	37.45 ± 4.39 a	26.48 ± 2.73 b	28.79 ± 2.66 b	20.97 ± 0.39 c

Different letters (a–e) on the same line represent a significant difference (*p* < 0.05).

**Table 2:** Particle size distribution, colors, and adsorption properties of JRDF, JRDF-E, JRDF-EPG, and JRDF-EAG.

Properties		JRDF	JRDF-E	JRDF-EAG	JRDF-EPG
D_3,2_ (μm)		116.47 ± 4.71 b	76.71 ± 2.07 d	59.38 ± 3.35 e	95.23 ± 4.05 c
Surface area (m^2^∙kg^−1^)		64.53 ± 3.74 d	105.15 ± 4.74 b	167.35 ± 4.42 a	86.44 ± 2.95 c
L*		53.8 ± 1.02 a	40.75 ± 3.13 b	36.95 ± 2.44 b	38.56 ± 3.34 b
*a* *		7.95 ± 0.23 b	10.57 ± 0.32 a	11.75 ± 0.37 a	9.36 ± 0.27 a
*b* *		11.32 ± 0.26 c	14.26± 0.26 b	19.54 ± 0.37 a	15.08 ± 1.42 b
Δ*E*		Control	13.63 b	19.13 a	15.76 a b
Water-retention ability (g∙g^−1^)		6.68 ± 0.24 c	7.17 ± 0.36 b c	10.96 ± 0.49 b	12.84 ± 0.37 a
Water-swelling volume (mL∙g^−1^)		6.05 ± 0.04 c	8.42 ± 0.16 b	8.80 ± 0.34 b	10.80 ± 0.26 a
Viscosity (cP)		2.01 ± 0.27 c	5.56 ± 0.08 b	7.00 ± 0.44 b	9.37 ± 0.24 a
Oil sorption ability (g∙g^−1^)		0.78 ± 0.09 c	1.24 ± 0.11 b c	3.73 ± 0.23 a	1.89 ± 0.03 b
Sodium cholate sorption ability (g∙g^−1^)		10.78 ± 0.46 c	10.27 ± 2.21 c	30.86 ± 1.24 a	20.46 ± 1.85 b
Glucose sorption ability (μmol∙g^−1^)	pH 2.0	6.49 ± 0.26 c	14.43 ± 0.36 a	9.26 ± 1.02 b	13.75 ± 0.45 a
	pH 7.0	10.17 ± 0.38 c	24.94 ± 2.02 a	14.52 ± 0.52 b	21.69 ± 1.17 a
NO^−^ sorption ability (μg∙g^−1^)	pH 2.0	7.36 ± 0.38 d	11.43 ± 0.23 c	21.64 ± 1.95 a	16.54 ± 1.25 b
	pH 7.0	2.48 ± 0.08 c	6.32 ± 0.05 b	16.85 ± 0.41 a	9.69 ± 0.32 b
Equilibrium sorption amount of Cu^2+^ (mg∙g^−1^)		9.58 ± 0.25 c	14.54 ± 0.43 b	19.62 ± 1.37 a	21.41 ± 0.27 a
Equilibrium sorption amount of Pb^2+^ (mg∙g^−1^)		13.42 ± 0.35 c	20.45 ± 0.75 b	18.32 ± 0.85 b	25.64 ± 1.22 a

Different letters (a–e) on the same line represent a significant difference (*p* < 0.05).

## Data Availability

The data presented in this study are available on request from the corresponding author. The data are not publicly available due to the data are contained within the article.
